# The Influence of Potassium Salts Phase Stabilizers and Binder Matrix on the Properties of Novel Composite Rocket Propellants Based on Ammonium Nitrate

**DOI:** 10.3390/ma15248960

**Published:** 2022-12-15

**Authors:** Traian Rotariu, Bogdan-Gheorghe Pulpea, Florin-Marian Dîrloman, Aurel Diacon, Edina Rusen, Gabriela Toader, Neculai-Daniel Zvîncu, Tanţa-Verona Iordache, Răzvan Horia Botiș

**Affiliations:** 1 Military Technical Academy “Ferdinand I”, 39-49 George Coșbuc Boulevard, 050141 Bucharest, Romania; 2 Faculty of Chemical Engineering and Biotechnologies, University Politehnica of Bucharest, 1-7 Gh. Polizu Street, 011061 Bucharest, Romania; 3 National Institute of Research and Development for Chemistry and Petrochemistry, 202 Splaiul Independentei, 060041 Bucharest, Romania; 4 National Romanian Company ROMARM S.A., 5 Timișoara Boulevard, 061301 Bucharest, Romania

**Keywords:** phase-stabilized ammonium nitrate, polymorphism, rocket propellants, binder, polyurethane

## Abstract

The environmental impact and availability of ingredients are vital for the new generation of rocket propellants. In this context, several novel composite propellants were prepared based on the “greener” oxidizer *phase-stabilized ammonium nitrate* (PSAN), a micronized aluminum–magnesium alloy fuel, iron oxide powder burn rate modifier, triethylene glycol dinitrate (TEGDN) energetic plasticizer and a polyurethane (PU) binder. The novelty of this study is brought by the innovative procedure of synthesizing and combining the constituents of these heterogeneous compositions to obtain high-performance “eco-friendly” rocket propellants. The polymorphism shortcomings brought by ammonium nitrate in these energetic formulations have been solved by its co-crystallization with potassium salts (potassium nitrate, potassium chromate, potassium dichromate, potassium sulphate, potassium chlorate and potassium perchlorate). Polyester–polyol blends, resulting from recycled post-consumer polyethylene terephthalate (PET) glycolysis, were utilized for the synthesis of the polyurethane binder, especially designed for this type of application. To adjust the energetic output and tailor the mechanical properties of the propellant, the energetic plasticizer TEGDN was also involved. The performance and safety characteristics of the novel composites were evaluated through various analytical techniques (TGA, DTA, XRD) and specific tests (rate of combustion, heat of combustion, specific volume, chemical stability, sensitivity to thermal, impact and friction stimuli), according to NATO standards, providing promising preliminary results for further ballistics investigations.

## 1. Introduction

In the area of explosive formulations (propellants, explosives and pyrotechnics), the crystalline oxidizers supply the necessary amount of oxygen for the rapid and exothermic combustion of a fuel, selected from organic compounds (binders) and/or metallic powders (aluminum, magnesium, boron, etc.).

Historically, the first oxidizer used in the development of heterogeneous mixtures was the Indian saltpetre, known nowadays as potassium nitrate. Its applicability at that time was limited to the development of the well-known black powder (mechanical mixtures based on 75% potassium nitrate, 15% charcoal and 10% sulfur), as a bursting explosive substance as well as a gun and rocket propellant [1,2]. Over time, the number of oxidizers involved in energetic mixtures rapidly increased, including other compounds, such as perchlorates, chlorates, peroxides, sulphates, chromates, dichromates, polyhalogenated compounds, etc., enlarging the area of applicability [1,2,3,4].

In recent decades, ammonium perchlorate (AP), an oxidizer from the perchlorate class, has been the preferred oxidizer for the area of solid composite propellants. It is considered optimal for use in rocket motors [1,2,3,4,5,6,7] due to its availability, high oxygen balance, good thermal stability and compatibility with other components. This versatile oxidizer, embedded into a polyurethane matrix (mostly HTPB, hydroxyl-terminated polybutadiene), has proven its applicability from ballistic missile systems used in the tactical field, to rocket motors used to ensure the propulsion of spacecrafts in the atmosphere [2,3,5]. In addition to this heterogeneous composite rocket propellant, it is worth mentioning the double-base (DB) propellants. Colloidal mixtures based on nitrocellulose plasticized with nitroglycerine and small amounts of additives have been frequently used in military applications (primarily in ballistic missiles). However, for safety reasons, DB utilization has declined over time. Analyzing the AP oxidizer, although it possesses energetic advantages, the regulations governing the preservation of the environment and the individuals raise doubts about its viability in the near future, due to the high toxicity of the perchlorate anion, combined with the ozone depletion action of the hydrochloric acid released in the atmosphere. Therefore, it is necessary to reduce its use or replace it with alternative oxidizers that present both the “greener” character and the low vulnerability ammunition (LOVA) characteristic, while maintaining acceptable performances. Low vulnerability is another aspect that is currently being emphasized, along with green chemistry, to reduce the probability of accidental ignition during the entire life span of the ballistic system. The most promising candidates for crystalline “greener” oxidizers are ammonium dinitramide (ADN), hydrazinium nitroformate (HNF), hydroxyl–ammonium nitrate (HAN) and ammonium nitrate (AN) [5,8,9,10,11,12,13]. Due to its superior energetic performance compared to AP, ADN is one of the most targeted alternatives to be used in the development of this type of energetic mixture [5]. However, aspects related to its production, such as compatibility with diisocyanates, high hygroscopicity and increased sensitivity to solar light, limit its applicability. HNF and HAN, similar to ADN, are substances that are difficult to produce and exhibit poor compatibility when added to polyurethane-based compositions. In this context, the alternative that has received a significant amount of interest in this field of research is AN. AN is a “greener” chlorine-free oxidizer, highly accessible, which is widely used in the agricultural field as a fertilizer and that does not have harmful effects on the environment and the population [5,12,13,14]. However, there are some objections to its applicability in composite rocket propellants, which are related to its performance but especially to the crystalline-phase transitions that can occur even at ambient temperature. The phase transitions of the AN crystal at ambient temperatures pose serious problems for its use in polymeric composites as the variations in the volume of the crystals produce cracks in the binder matrix, after just a few thermal cycles. These cracks will not only affect the mechanical properties of the charge but can seriously alter the geometry of the propellant grain and lead to the catastrophic failure of the engine. Several studies have reported that this polymorphism can be overcome by using other compounds, capable of leading to the disappearance of these crystal changes [5,12,13]. Alternatively, energetic performances could be enhanced by using a more energetic metallic fuel (magnesium, boron) or an energetic binder (glycidyl azide polymer) [5,9,10,11]. Additionally, some formulations found in the literature still contain certain proportions of AP along with AN oxidizer, to maintain the acceptable performance or to limit the introduction of active metallic fuels (such as magnesium powders), which could lead to chemical instability for long-term storage. The consecrated polymeric binders employed in composite rocket propellants also pose problems due to their lack of elasticity or toxicity brought by the solvents employed along with them. One of the key objectives is to add phase-stabilized AN into rocket propellant formulations while overcoming the drawbacks of traditional polymeric binders. Considering that the combustion of a propellant takes place in a very controlled manner, at a well-defined speed, the cracks that may appear at the level of the grain could lead to uncontrolled combustion, which in most cases concludes with the destruction of the system and the injury of the operating personnel.

In this paper, a new group of “greener” oxidizers synthesized by the co-crystallization of ammonium nitrate with various potassium salts were prepared. These phase-stabilized ammonium nitrate formulations were subsequently used as oxidizers in various rocket propellant composites, comprising an eco-friendly polyurethane binder originating from polyols obtained through the recycling of PET waste, a fine metallic fuel (aluminum–magnesium powder), an energetic plasticizer (triethylene glycol dinitrate, TEGDN) and a catalyst (iron oxide, Fe_2_O_3_).

Therefore, the novelty of this study is represented by the innovative procedure of combining the constituents of these heterogeneous compositions to obtain high-performance, “eco-friendly” rocket propellants. The polymorphism shortcomings brought by ammonium nitrate in these energetic formulations have been solved by the co-crystallization with potassium nitrate, potassium chromate, potassium dichromate, potassium sulphate, potassium chlorate and potassium perchlorate. Polyester–polyol blends, resulting from recycled post-consumer polyethylene terephthalate (PET) degradation, were utilized for the synthesis of the polyurethane binder, especially designed for this type of application. Furthermore, the experimental data regarding the safety and performance characteristics evaluated through various analytical techniques (TGA, DTA, XRD) and specific tests (rate of combustion, heat of combustion, specific volume, chemical stability, sensitivity to thermal, impact and friction stimuli), according to NATO standards, provided a well-founded starting point for upcoming ballistic investigations [15,16,17,18,19].

## 2. Materials and Methods

### 2.1. Materials

#### 2.1.1. Materials for Phase-Stabilized Ammonium Nitrate

Ammonium nitrate (NH_4_NO_3_, 99%, Honeywell Fluka™, Seelze, Germany) and co-crystallizers for ***phase-stabilized ammonium nitrate*** (stabilizer “agents”, **SA**): potassium nitrate (KNO_3_, 99%, Honeywell Fluka™, Seelze, Germany), potassium chromate (K₂CrO₄, 99%, Honeywell Fluka™, Seelze, Germany), potassium dichromate (K_2_Cr_2_O_7_, 99%, Sigma Aldrich, St. Louis, MO, USA), potassium sulphate (K_2_SO_4_, 99%, Sigma Aldrich, St. Louis, MO, USA), potassium chlorate (KClO_3_, 99%, Honeywell Fluka™, Seelze, Germany), potassium perchlorate (KClO_4_, 99%, Honeywell Fluka™, Seelze, Germany) and distilled water (Honeywell Fluka™, Seelze, Germany) were used as received.

#### 2.1.2. Materials for “Greener” Composite Rocket Propellants

The above-mentioned synthesized phase-stabilized ammonium nitrate formulations were employed as oxidizers for the new “greener” rocket propellant composites. Polyester–polyol blends resulted from post-consumer polyethylene terephthalate (PET) degradation—according to ref. [20], Sethatane D1160 (**SET**, hydroxyl content—5.4%, Allnex, Brussels, Belgium), diphenylmethane–4,4′–diisocyanate (**MDI**, -NCO content—31.5% (weight %), technical product Desmodur^®^ 44V20L (Covestro, Leverkusen, Germany), triethylene glycol dinitrate (TEGDN)—an energetic plasticizer synthesized at MTA according to ref. [21], were vacuum dried for 24 h at 50 °C before being employed in polyurethane synthesis. As metallic fuel, an aluminum–magnesium alloy powder, with an average particle size <5 μm (**PAM**, Sigma-Aldrich, St. Louis, MO, USA) was used as received. As catalyst, iron oxide powder, with an average particle size of 50 μm (Fe_2_O_3_, Sigma-Aldrich, St. Louis, MO, USA), was used as received.

### 2.2. Methods

#### 2.2.1. Synthesis of Phase-Stabilized Ammonium Nitrate by Co-Crystallization Process

**Scheme 1:**
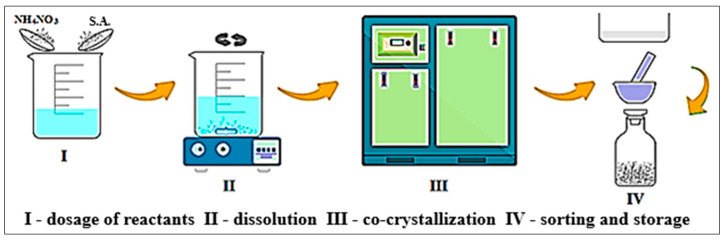
Flow chart of the synthesis of phase-stabilized ammonium nitrate. (S.A.—stabilizing agent).

The “green” oxidizer formulations (referred to as PSAN_0, PSAN_1, PSAN_2, PSAN_3, PSAN_4, PSAN_5 and PSAN_6, according to the compositions described in Table 1), key ingredients for the synthesis of our “eco-friendly” propellants, were obtained through the following three main steps: In the first step, a concentrated solution was prepared by dissolving 90 g of ammonium nitrate in 200 mL of deionized water. In the second stage, 10 g of stabilizing agent (SA) was added to the solution, under continuous stirring, at 25 °C, until complete dissolution. The final aqueous solutions was vacuum dried (for 72 h at 50 °C) and distinct phase-stabilized ammonium nitrate batches were obtained (their composition is displayed in Table 1. The scheme of the laboratory-scale synthesis of PSAN is described in Scheme 1).

#### 2.2.2. The Processing of the Composite Rocket Propellant

**Scheme 2:**
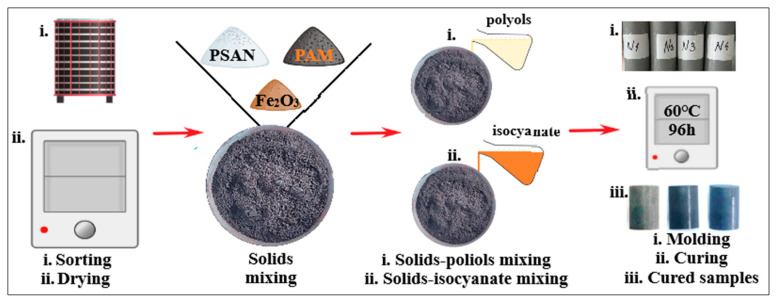
Flow chart for producing composite propellants.

The composites employed as rocket propellants are heterogeneous mixtures comprising granular materials (oxidizers, metallic fuels and additives) embedded in a polymeric matrix. Modern rocket propellants are manufactured using cast and cure processes as they are based on polyurethane binders. In this work, a similar process was employed at the laboratory scale. The technology started with the dry mixing of the solid components (oxidizer, metallic fuel, catalyst) followed by a wet mixing with the organic components (blends of polyurethane precursors: plasticizer, MDI, SET and polyester–polyol). For this purpose, the oxidizer (PSAN) was previously granulometrically sorted and vacuum dried, then mixed with the metallic fuel and the catalyst, until complete homogenization of the solids. In the wet mixing phase, the solids were first mixed with the polyols and the energetic plasticizer, and then the aromatic diisocyanate was introduced in the final stage of the process. The wet mixtures obtained were promptly cast in cylindrical molds and allowed to cure in a vacuum oven, for 96 h, at 60 °C. The procedure described above can be better understood in conjunction with the illustrations presented in Scheme 2, while the propellant formulations are summarized in Table 2.

#### 2.2.3. Characterization

The polymorphic behavior of the synthesized PSAN formulations was investigated by TGA analysis. The thermogravimetric analysis (TGA) of the salts was executed using a Netzsch TG 209 F3 Tarsus instrument (NETZSCH, Selb, Germany). The experiments were performed at a heating rate of 10 °C/min, under nitrogen flow. Samples of approximately 4 mg were heated from 25 °C to 900 °C. The formulations were also characterized by X-ray diffraction (XRD) using a Bruker D8 Advance XRD (Karlsruhe, Germany). Each sample was scanned in the angular range 2θ, 10°–60° with a sampling interval of 0.2°. XRD reflections were obtained at ambient temperature (25 °C). The particle size for the crystalline materials during investigations was 100 μm.

To examine the behavior of the synthesized propellants when subjected to high thermic variations (heating–cooling cycles), a climatic chamber (Binder ED 115) was used under high vacuum conditions. The structural integrity of the PSAN samples was studied in various thermal regimes. The temperature varied from −5 °C to +100 °C. Each cycle consisted of maintaining the samples at −5 °C for 12 h, then increasing the temperature to +100 °C and maintaining it for 12 h. The heating and cooling rate was in all situations approximately 5 °C/min (±0.2 ng). The thermal properties of the propellants developed in this study were also investigated using DTA [19]. The differential thermal analysis system DTA OZM 551 Ex (OZM Research, Hrochův Týnec, Czech Republic) with Meavy dedicated software, version 2.2.5.43, was involved. During the investigation, 30 mg samples, with 100 μm granulation, were heated from 25 °C to 300 °C. The heating rate of the apparatus was 5 °C/min. The safety characteristics of the “greener” propellant formulations were assessed by measuring the friction sensitivity and impact tests. The friction sensitivity was determined using a BAM friction apparatus according to STANAG 4487 [18]. Thus, 30 tests were performed for each sample with various loading forces. The impact behavior was determined on a KAST Hammer apparatus (Julius Peters, Berlin, Germany), where 30 tests were performed for each sample, according to STANAG 4489 [15]. An IKA adiabatic calorimeter and a Julius-Peters gas meter (IKA^®^-Werke GmbH & Co. KG, Staufen, Germany), were used to estimate the heat of combustion and the specific volume for 2 g samples of each energetic formulation, having an average particle size of 500 μm. Duplicate tests were carried out for each composite formulation under vacuum conditions and average values were reported with an error of ±5%. Vacuum stability tests (VSTs) were conducted according to STANAG 4556 [16] with a STABIL apparatus, where 5 g of sample was heated at 100 °C, and maintained at this temperature for 40 h, under vacuum. The volume of the gas released was recorded by a pressure transducer connected to a computer. A FLIR thermal camera was used to investigate the combustion mechanism in atmospheric conditions. Cylindrical specimens 15 mm in diameter and 30 mm in length were employed for the combustion experiments.

## 3. Results and Discussion

**Figure 1:**
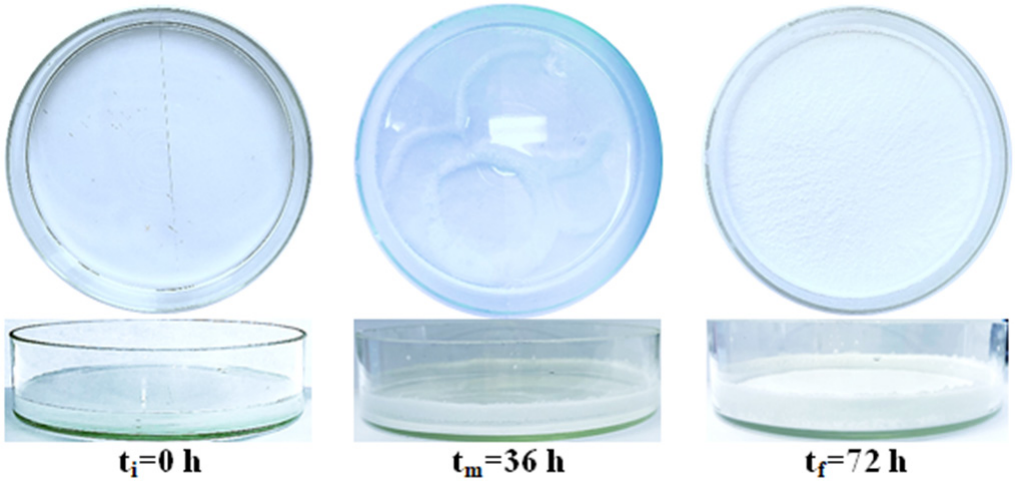
The co-crystallization process for PSAN_0 at different stages.

**Figure 2:**
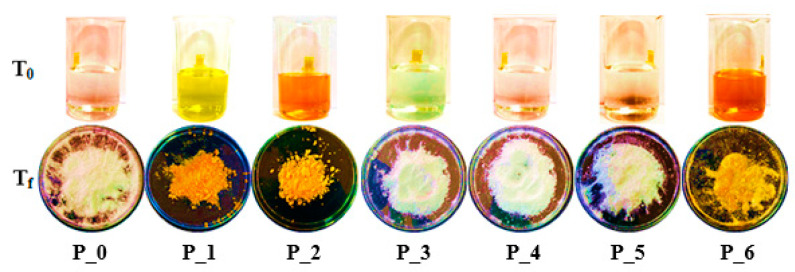
PSAN synthesized formulations (**T_0_**—co-crystallization initial time; **T_f_**—co-crystallization final time).

Based on our previous study [22], the co-crystallization of AN with various potassium salts determined hydrogen bonds due to the polar groups with ammonium ions. This led to the inhibition of the polymorphic transformations occurring in the temperature range from −5 to + 100 °C. The duration of the co-crystallization process is strictly dependent on the volume of each sample resulting from the dissolution process. For instance, in our case, for a mixture consisting of 90 g NH_4_NO_3_, 10 g KNO_3_ and 200 mL of distilled water, the time required was 72 h, resulting in a final quantity of approximately 95 g of PSAN (95% yield). The co-crystallization process, at various time intervals, is illustrated in Figure 1, while the PSAN co-crystals are shown in Figure 2.

**Figure 3:**
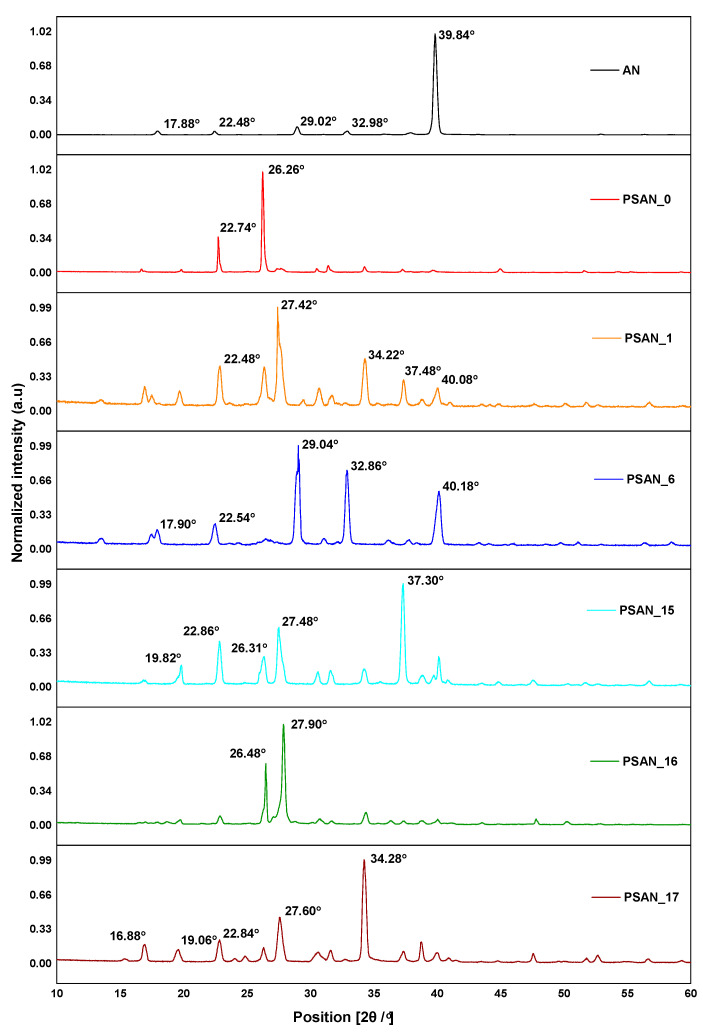
XRD profiles of the PSAN formulations.

The results obtained by the XRD analysis highlighted that the stabilizing additives modified the AN crystalline structure, according to the variation of the intensity and angular positions of the peaks. The XRD profiles for the analyzed samples are given in Figure 3. Although the crystalline structure of ammonium nitrate was modified, it could not be confirmed that the phased-stabilized ammonium nitrate was in phase II [23]. Therefore, the stabilized ammonium nitrate formulations were subjected to XRD analysis to evaluate the ammonium nitrate crystals mixed with the stabilizer additive. For ammonium nitrate in pure state, the peaks appear at 17.88°, 22.48°, 29.02°, 32.98° and 39.84°. Thus, comparing the recorded data with those from the literature [23], the ammonium nitrate used in the synthesis is in phase IV, orthorhombic, of polymorphic transformation at ambient temperature [24,25]. Based on the information presented in Figure 3, it can be observed that the resulting peaks are similar, but noticeable differences appear in the case of PSAN_0 and PSAN_16, where most of the peaks are attenuated and the values for phase III vary. As for PSAN_1, PSAN_4 and PSAN_6, the variations appear mainly for phase III, where the diffraction peak values vary, at 27.42°, 27.48° and 27.60°, respectively. Although PSAN_2 contains potassium chloride, the results obtained are similar to those of AN, as confirmed by previous investigations.

**Figure 4:**
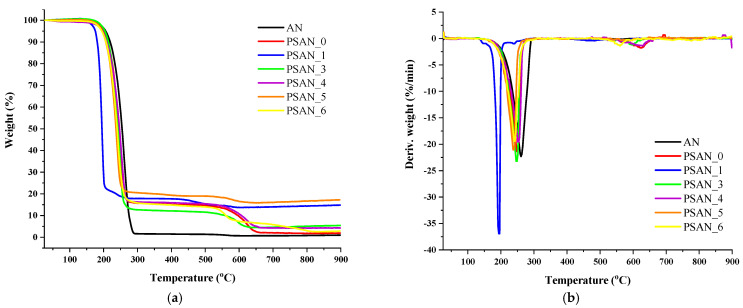
Multigraph with TGA (**a**) and DTG (**b**) thermograms for the PSAN specimens.

According to the literature data, several thermal parameters are relevant for the phase-stabilized ammonium nitrate decomposition behavior, such as thermal decomposition temperature intervals, decomposition onset temperature, maximum exothermic temperature and weight/mass loss [23]. Thus, to obtain complementary information regarding the influence of the stabilizing agents on the thermal decomposition behavior of the composite propellants, the synthesized PSAN formulations were subjected to thermogravimetric analysis. Figure 4 displays TGA (a) and DTG (b) curves for the synthesized PSAN formulations. As can be observed in Figure 4 and Table 3 the decomposition process of the samples began approximately at the same temperature, except PSAN_1 (based on potassium chromate), which began considerably earlier, at 200 °C, where it lost 75.12 wt.%. This aspect can also be observed in Table 4, where the maximum decomposition temperatures, corresponding to the maximum of the DTG peaks, are summarized. Additionally, the peaks displayed in the DTG profile (see Figure 4b) offer data about the succession of the degradation stages of the PSAN formulations. The maximum of the DTG peak attributed to PSAN_1 was observed at 181.21 °C, followed by PSAN_16, at 206.86 °C, PSAN_6, at 207.47 °C and PSAN_4, at 208.88 °C. The other formulations (PSAN_0 and PSAN_3) seem to start decomposition around 213 °C. Based on the recorded data, the pure-state ammonium nitrate decomposed completely at 288.77 °C when its mass was reduced by 98.17 wt.%. The decomposition peaks for the PSAN formulations were strictly dependent on the decomposition points of each potassium salt.

The information regarding the degradation stages of PSAN decomposition is presented in Table 3 and Table 4. It can be observed that the mass loss in the interval 200–300 °C is lower for all PSAN samples compared to pure AN, due to the formation of potassium oxide/salts which then suffer further decomposition at higher temperatures, or remain at an almost constant mass (like in the case of PSAN_1 and PSAN_5). Although PSAN_1 started to decompose earlier than the other formulations, at the end of the survey (900 °C), it lost only 86.86 wt.% of its initial weight. The same aspect was also observed for PSAN_5, which presented the smallest percentage of mass loss of 84.22 wt.%.

**Figure 5:**
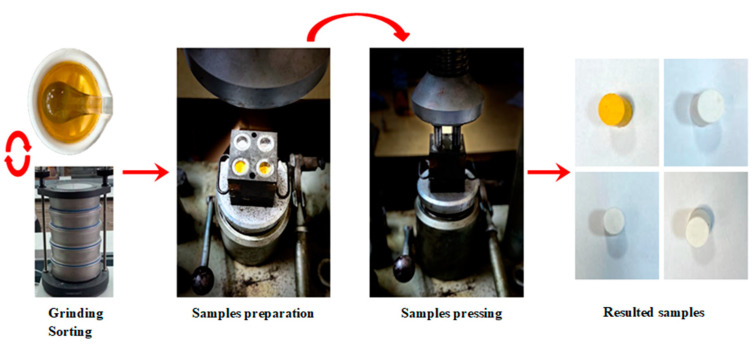
Technological process of PSAN cylindrical formulations.

To further investigate the effectiveness of the stabilizing additives, each sample was subjected to a series of thermal cycles (as described in the Methods section). Samples of 25 g of stabilized ammonium nitrate with an average particle size of 100 μm were placed in a mold (with ϕ = 18.60 mm) and pressed using a hydraulic press. Cylindrical specimens with heights of 18.80 mm were formed, as presented in Figure 5. The compressive force applied was 15 bars.

**Figure 6:**
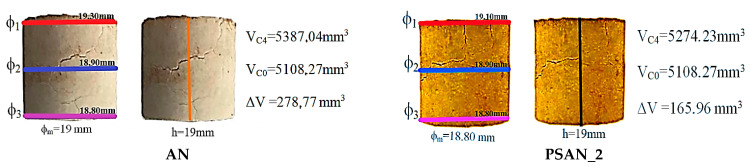
Volume variation for the sample containing neat AN and PSAN_6 after four heating–cooling cycles (**V_C4_**—volume after cycle “4”; **V_C0_**—initial volume).

**Figure 7:**
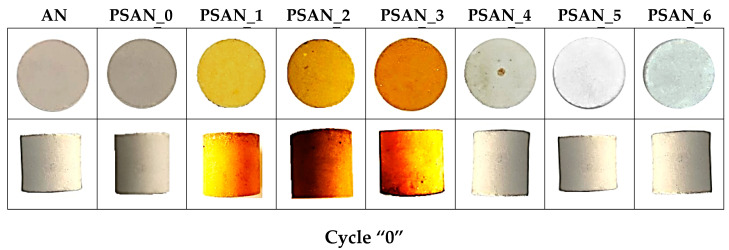

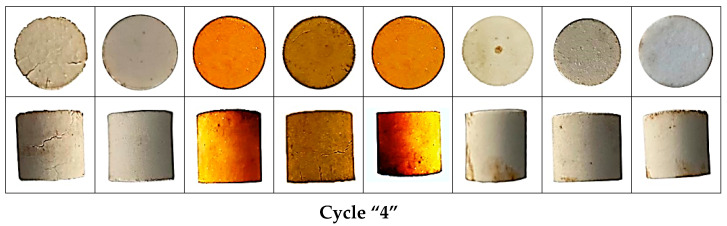
Comparison of morphological integrity of PSAN cylinders after being subjected to four thermal cycles.

The thermal cycle investigations were conducted until noticeable cracks could be observed on the surface of the cylinders. The data collected after the heating–cooling cycles revealed that the additives with potassium in their structure were particularly successful at preventing polymorphic transitions, in the interval –5 °C–+100 °C, except for potassium dichromate (K_2_Cr_2_O_7_). The phase transition of the co-crystalized oxidizer inside these samples led to the appearance of imperfections in the material, producing variations in the volume of the probes. For example, in the case of a cooling phase from +100 °C to –5 °C, the volume slightly decreased when a phase transition occurred. Consequently, a crack appeared in the structure of the sample. When calculating the volume variation, an increase of 5.2% was obtained when employing neat ammonium nitrate and 4.5% for PSAN_2. An average value was established by measuring the diameter of three parallel cross-sections (ϕ1, ϕ2, ϕ3), as illustrated in Figure 6. The effects of the fourth temperature cycle on the PSAN-synthesized formulations are displayed in Figure 7.

Through the variation of grain size, grain shape and chemical nature of the oxidizer and metallic fuel particles, the following properties can be tailored or enhanced: loading density, thermal stability, burning rate or mechanical properties. Furthermore, additional information can be provided on how these characteristics and the interactions between the particles and polymer matrix interrelate.

The theoretical and experimental evaluation of physical, chemical and thermodynamic properties represents the first step in the development of an energetic material. Safety and performance characteristics generally depend on several parameters, including physical properties (structural configuration, loading density), thermodynamic properties (heat of combustion, temperature and rate of combustion, specific volume), sensitivity (thermal, impact, friction, self-ignition) and chemical stability (compound compatibility) [17].

**Figure 8:**
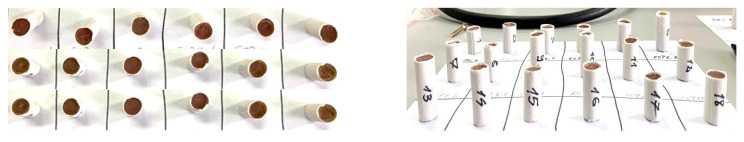
Cylindrical grain propellant molds.

The loading density represents the ratio between the amount of the composition (m) and its volume (V). To determine this parameter, composite mixtures were cylindrically shaped, with a diameter of 15 mm and a height of 30 mm. The obtained cylinders of composite material were sized to confirm the specified diameter and height, and subsequently weighed to determine the density. The calculated values are shown in Table 5, while in Figure 8, the cylindrical configurations of the propellants from the technological process are given.

Based on the calculated data, it seems that the density variation occurs due to the class of stabilizer used as well as due to the energetic plasticizer. TEGDN reduces the viscosity of the polyurethane, thus enhancing the propellant processability. Therefore, the highest loading density was obtained for the GP130 sample, containing 4.5% TEGDN and potassium chromate (K_2_CrO_4_)-stabilized ammonium nitrate (PSAN_1).

**Figure 9:**
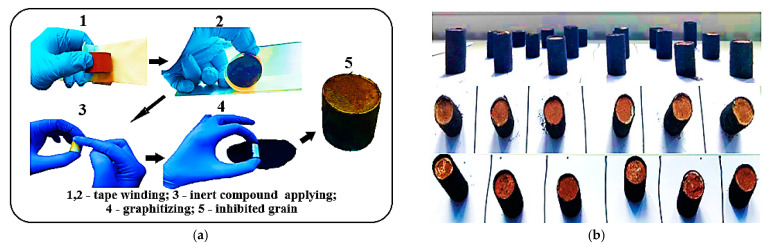
Inhibition process of cylindrical grain propellants. (**a**) Inhibition stages; (**b**) inhibited samples.

The burning rate and flame profile depend on the composition, structural homogeneity and loading density of the propellant mixture. As a result, the combustion behavior depends on the type, amount and distribution of the oxidizer, metallic fuel and binder. The evaluation of the burning mechanism was conducted at ambient pressure, with frontal combustion of the formulations, as presented in Figure 9. The samples were inhibited on the outer surface to achieve a neutral frontal burning, where the process will take place in parallel layers, in which case the combustion surface is considered to be constant.

**Figure 10:**
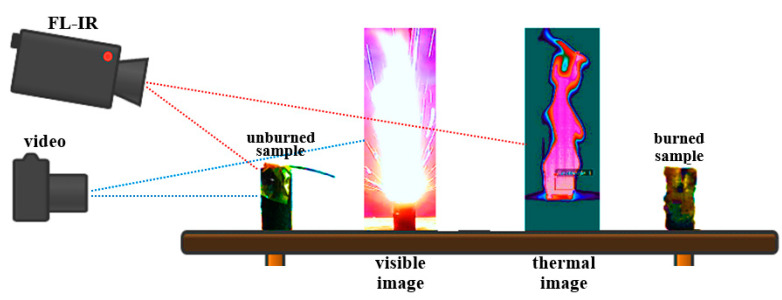
Setup for the burning tests at atmospheric pressure.

The ignition of the samples was performed with primer composition based on potassium nitrate, aluminum–magnesium alloy and binder, placed on the flat surface of the specimen, according to the setup described in Figure 10.

**Figure 11:**
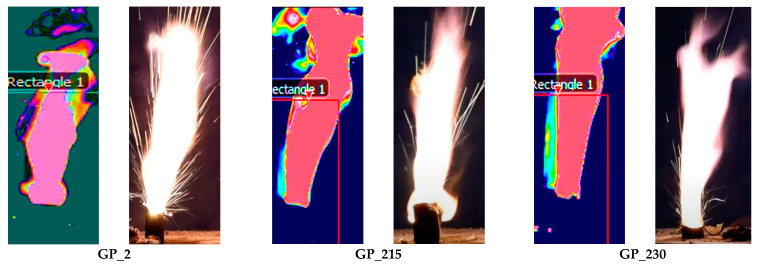
Flame configuration for rocket propellant formulations.

Due to the fact that the combustion of the propellant grain took place in a neutral regime, the linear burning rate was assessed by calculating the ratio between the height of the cylindrical sample and the burning time. To see whether the data were accurate, the burn rate was also verified based on the temperature profiles obtained with the thermal device (see Figure 11). The results for the burning rate are shown in Table 6, and the flame profiles for the formulations of composite rocket propellants based on PSAN_3 (NH_4_NO_3_:KNO_3_:K_2_Cr_2_O_7_) can be visualized in Figure 11. During the investigation, the burning rate increased with the amount of energetic plasticizer added to the composition. Thus, the stabilizing compounds did not show a significant impact on this parameter at ambient pressure.

**Figure 12:**
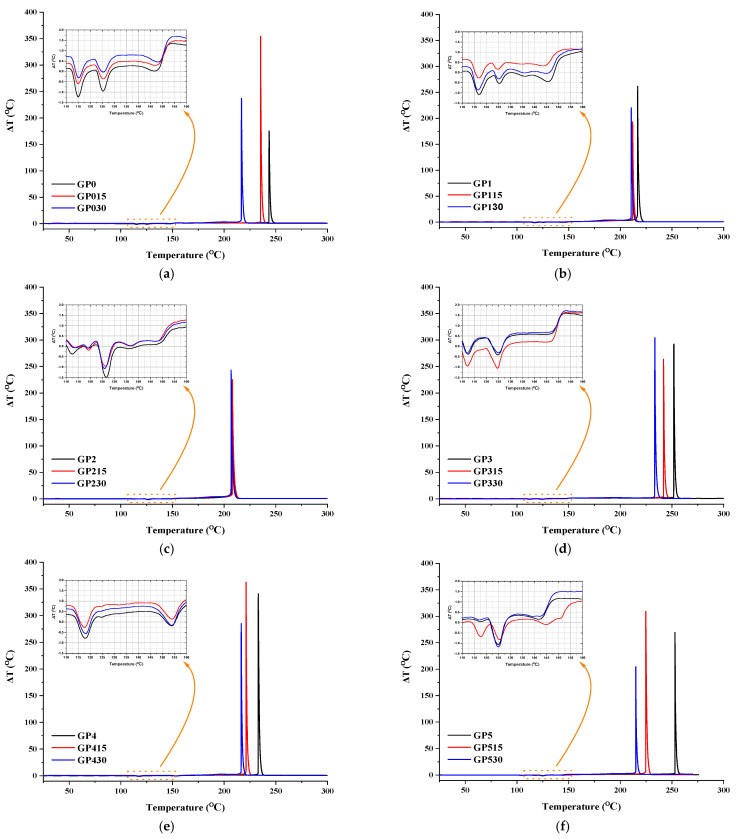
DTA thermograms of “greener” composite rocket propellants. (**a**) PSAN _0; (**b**) PSAN_1; (**c**) PSAN_3; (**d**) PSAN_4; (**e**) PSAN_5; (**f**) PSAN_6).

DTA analysis of the rocket propellants showed that their sensitivity to thermal stimulus is strictly dependent on chemical composition. Thus, for samples containing 4.5% TEGDN, the obtained values are slightly lower than those containing 2.25% TEGN. Focus has therefore been placed on their thermal behavior without the prior addition of the energetic plasticizer because the main purpose of this research was to examine the impact of potassium salts in composite formulations. Composite rocket propellants without TEGDN show higher thermal sensitivity, except for GP2 (containing potassium nitrate <KNO_3_> and potassium dichromate <K_2_Cr_2_O_7_>). These possess a similar thermal behavior to TEGDN-containing formulations. It can be stated that the use of potassium dichromate led to this thermal behavior, observable also in cases of potassium chromate (K_2_CrO_4_)-containing mixtures, with values close to mixtures with TEGDN. Overall, the temperature sensitivity of other samples is above 220 °C. Therefore, it can be concluded that the temperature sensitivity of the developed mixtures is acceptable (with values higher than 200 °C). It can also be highlighted that the composite formulations undergo the same three endothermic transformations that correspond to the PSAN phase transition, as per in details from Figure 12. Polymorphic transformations of the oxidizers can be observed at values of 110 °C, 122 °C and 145 °C. These values are far from the temperatures encountered in the environment. The DTA results are illustrated in Figure 12 and Table 7.

The evaluation of the combustion heat (Q_v_) and specific volume (V_sp_) of the mixtures was conducted using a calorimetric bomb (closed vessel) coupled with a gas meter. Thus, 2 g samples for each composite mixture were ignited in the 25 cm^3^ calorimetric bomb. The generated gases were cooled at room temperature and expanded in a vacuumed gasometer having a volume of 3180 cm^3^. Based on the temperature and pressure variations, the heat of combustion and specific volume were calculated using Equations (1) and (2), respectively. For this investigation, cured rocket propellants were cut and sieved to obtain grains with an average diameter of 500 μm. Duplicate tests were performed for each formulation.

(1) $$Q_{v}=\frac{K\times \Delta t-q}{\omega }$$

where:

K—caloric equivalent of the apparatus, (KJ/°C);
Δt—temperature variation measured, (°C);
q—combustion heat produced by the ignition wire, (KJ);
ω—sample amount, (g);

(2) $$V_{\mathrm{sp}}=\frac{W\times \Delta P\times 273.15}{\omega \times 760\times \left(273.15+t\right)}$$

where:

W—flask volume + calorimeter bomb volume, [l];
ΔP—pressure variation (mercury column height) (mmHg);
ω—sample amount, (Kg);
t—ambient temperature, (°C).

The heat of combustion is one of the main performance characteristics of rocket fuels. A high heat of combustion often leads to a high temperature of the combustion products and subsequently to a higher pressure and specific impulse. Based on the data from Table 8, it can be seen that the caloric output of composite propellant mixtures based on different PSAN formulations is lower than typical compositions with ammonium perchlorate. However, it is comparable to double-base propellants (propellants containing nitrocellulose and nitroglycerin) [2,3]. On the other hand, the specific volume is much higher for PSAN-based mixtures, and this could partially compensate for the lower caloric output.

Taking into account only the influence of the stabilizing agent within the oxidizer, almost imperceptible differences are obtained for the heat of combustion and specific volume. An increase in combustion heat and a decrease in the specific volume with the introduction of the energetic plasticizers in the mixtures can also be noted.

The mechanical stimuli of “impact” and “friction” were applied to evaluate the safety features of the compositions during handling and transport. The variation of the data obtained is related to the constituents of the mixture and the stabilizers used. Friction sensitivity tests showed that composite materials are not sensitive to friction (giving reaction at maximum values of force applied). Samples GP1, GP2, GP3 and GP5 (with chromate, dichromate, chlorate and perchlorate, respectively) exhibited a sound type reaction. The use of TEGDN annihilated this behavior by softening the composition. A possible decomposition reaction could be considered for the rest of the samples by colorimetric analysis of the porcelain test plates. The friction sensitivity test results are given in Table 9.

**Figure 13:**
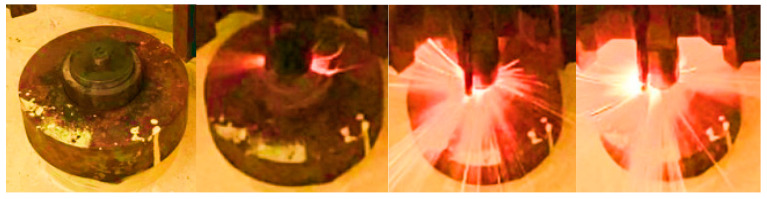
Reactions developed by the composite formulations during impact tests.

The impact tests were carried out for composite materials in dried powder form, with an average grain diameter of 500 μm. A 1 kg hammer was used and the height range was from 30 to 50 cm. The impact energy was calculated with Equation (3). It can be stated that the tested samples are rather sensitive to impact, having values in the range of 3–4 J. The results are presented in Table 10, while in Figure 13 some typical reactions are illustrated.

The chemical stability of the propellants over time is another important parameter to determine when considering their life span. Artificial ageing is continuously involved when evaluating this parameter. The vacuum stability method is preferred by NATO countries as it evaluates thermal stability of energetic materials by measuring the volume of gases released after ageing at a known temperature. The equation used to determine the volume of released gases (V_deg_), at a temperature of 273 K and a pressure of 10, has the following form:

(3) $$V_{\deg}=\left[V_{c}+V_{t}-\frac{m}{d}\right]\times \left[\frac{P_{2}\times 273}{t_{2}+273}-\frac{P_{1}\times 273}{t_{1}+273}\right]\times \frac{1}{1013}$$

where:

V_deg_—volume of gases released by the sample (cm^3^);
V_c_—volume occupied by the transducer and the adapter (cm^3^);
V_t_—volume of the heating tube (cm^3^);
m—sample amount (g);
d—density of the tested sample (g/cm^3^);
P_1_—initial pressure (bar);
P_2_—final pressure (bar);
t_1_—initial ambient temperature (°C);
t_2_—final ambient temperature (°C).

**Figure 14:**
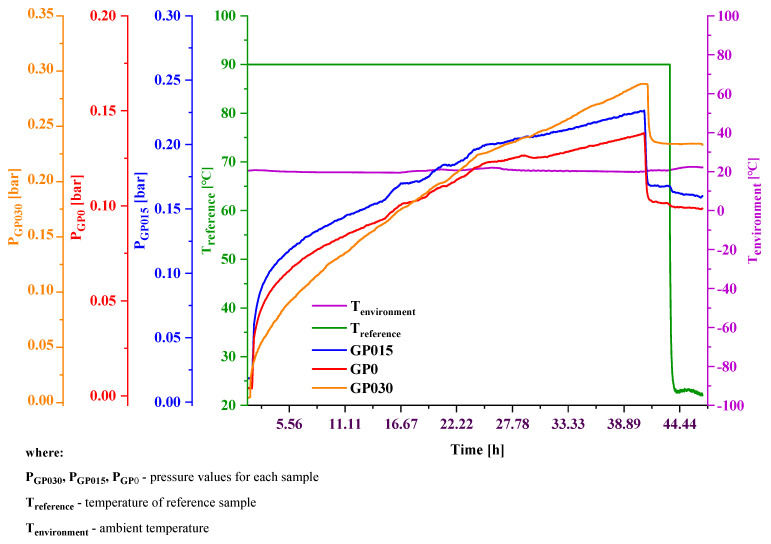
Temperature profiles for PSAN_0 based composite mixtures with various TEGDN contents.

The goal of the vacuum stability analysis was to establish whether the materials utilized to manufacture the solid rocket composite propellants are chemically compatible over time. According to STANAG 4556 [19], acceptable stability is considered for a determined volume of the sample not exceeding 2 cm^3^/g. Values obtained for the tested mixtures are below the standard threshold value. The addition of TEGDN, an energetic plasticizer from the nitro-ester class, led to an increase in the volume of volatile gases. Even so, the obtained values are in the compliance range. The calculated results are presented in Table 11. In Figure 14 are presented the temperature profiles for rocket propellants based on PSAN_0.

## 4. Conclusions and Perspectives

This study, regarding the development of novel solid rocket propellants, was focused on two research directions. Firstly, the effect of potassium salts on the crystalline structure of ammonium nitrate (the oxidizer) was studied through thermal analysis and X-ray diffraction. The X-ray diffraction analysis indicated the successful co-crystallization of AN with all six potassium salts. The effective phase stabilization of AN was demonstrated using the DTA analysis, further performed on propellant composites. In the temperature range of −5 to + 100 °C, no polymorphic transitions were observed for all six potassium salts, confirming the removal of the phase transitions IV-III of the oxidizer, while the phase transitions III-II were shifted up, over 100 °C. Thus, all the potassium salts improve the polymorphic behavior of ammonium nitrate at ambient temperatures.

This conclusion was also verified by comparing the morphological integrity of AN and PSAN-pressed grains (cylinders) after being subjected to four thermal cycles. Crack formation was observed for the AN grains and slight cracking was also observed for the co-crystal PSAN_2 (AN and K_2_Cr_2_O_7_).

The second research direction focused on the analysis of the new composite mixture formulations based on “eco-friendly” PSAN oxidizers, with various stabilizing agents that inhibit polymorphic behavior that occurs at ambient temperature. Their impact on the performance and safety characteristics of the propellants was determined by investigating the thermal and mechanical behaviors using various analytical techniques (DTA, vacuum thermal stability, impact and friction tests, heat of combustion and specific volume determinations, combustion tests in atmospheric conditions).

Besides confirming the phase stabilization of AN, the DTA analysis was used to study the thermal sensitivity of the propellants. For all the propellant samples, the thermal sensitivity was acceptable (onset temperature higher than 200 °C) and increased when the TEGDN energetic plasticizer proportion was higher. However, for the mixtures based on AN phase-stabilized with potassium dichromate (K_2_Cr_2_O_7_), the thermal sensitivity was higher (lower onset temperatures) and was not influenced by the presence of TEGDN.

The friction tests demonstrated that the analyzed materials were insensitive to friction (360 N), while the impact sensitivity was rather high, with values in the range of 3–4 J. The chemical stability is not influenced by polymorphic agents, and is instead dependent on the content of TEGDN energetic plasticizer used.

Vacuum stability tests indicated good chemical stability for all the new composite propellants, being smaller or equal to 1 cm^3^/g, with an increasing value in line with the increased TEGDN content.

The measurements regarding combustion velocity at ambient pressure showed that the stabilizing agents have no influence on this, while the burning rate strongly increased along with the amount of energetic plasticizer added to the composition (more than 40% for a 15% TEGDN content and more than 60% for 30% TEGDN content).

The tests regarding the performance of the propellants in terms of heat of combustion and specific volume indicated that there is an insignificant influence of the phase stabilizer agent for AN, but there is a strong dependence on the TEGDN content. As the TEGDN content increases, the heat of combustion increases and the specific volume decreases.

Comparative tests on the consecrated propellants AP/HTPB and DB enable us to conclude that the proposed rocket propellant composites, besides their environmentally friendly character, displayed better specific volume (up to 26% improvement) and inferior heats of combustion (17 to 48% lower) in comparison with consecrated AP/HTPB rocked propellants used in aviation missiles, and better heats of combustion (from 0 to 40% improvement) and inferior specific volume (from 22 to 32% lower) compared to DB propellants used in rocket propulsion.

Overall, the characterization of the formulations from the safety and performance point of view showed that their behavior is strictly dependent on the energetic components used in their manufacturing and that the phase stabilizers for AN have slight or no influence on these characteristics.

Based on the data obtained during this study, the composite heterogeneous mixtures (GP0, GP015 and GP030), based on PSAN_0 (KNO_3_ as a stabilizing “agent”), as well as due to the low toxicity and good energetic properties of the potassium salt, provide a good background for future ballistic investigations in terms of subscale motor and closed vessel testing for further performance characterization.

## Figures and Tables

**Table 1:** The composition of phase-stabilized ammonium nitrate formulations.

Sample	NH_4_NO_3_	KNO_3_	K₂CrO₄	K_2_Cr_2_O_7_	KClO_3_	K_2_SO_4_	KClO_4_
**PSAN_0**	✓	✓					
**PSAN_1**	✓		✓				
**PSAN_2**	✓			✓			
**PSAN_3**	✓	✓		✓			
**PSAN_4**	✓				✓		
**PSAN_5**	✓					✓	
**PSAN_6**	✓						✓

**Table 2:** “Green” rocket propellant composite formulations.

Sample	Compound Proportions [wt.%]							
	PSAN_0	PSAN_1	PSAN_3	PSAN_4	PSAN_5	PSAN_6	PU_31	TEGDN
**GP0**	72						15	
**GP1**		72					15	
**GP2**			72				15	
**GP3**				72			15	
**GP4**					72		15	
**GP5**						72	15	
**GP015**	72						12.75	2.25
**GP115**		72					12.75	2.25
**GP215**			72				12.75	2.25
**GP315**				72			12.75	2.25
**GP415**					72		12.75	2.25
**GP515**						72	12.75	2.25
**GP030**	72						10.5	4.5
**GP130**		72					10.5	4.5
**GP230**			72				10.5	4.5
**GP330**				72			10.5	4.5
**GP430**					72		10.5	4.5
**GP530**						72	10.5	4.5

* In addition, all samples contain 1% Fe_2_O_3_ (catalysts) and 12% PAM (metallic fuel).

**Table 3:** Decomposition of the PSAN formulations (weight loss with temperature increase).

Sample		Weight Loss								
		100 °C	200 °C	300 °C	400 °C	500 °C	600 °C	700 °C	800 °C	900 °C
**AN**	**[%]**	0	3.79	98.41	98.55	98.67	99.33	99.34	99.34	99.49
**PSAN_0**		0	4.79	84.34	84.93	85.4	90.42	98.01	98.22	98.41
**PSAN_1**		0	75.12	82.15	82.32	84.63	86.29	86.48	86.51	86.86
**PSAN_3**		0	4.24	87.36	87.87	88.47	93.65	95.43	95.44	95.49
**PSAN_4**		0	6.62	83.76	84.13	84.59	89.42	95.75	95.79	95.94
**PSAN_5**		0	7.49	79.47	80.64	81.04	83.49	83.97	83.98	84.22
**PSAN_6**		0	6.81	84.19	85.16	86.02	92.66	94.24	96.99	97.92

**Table 4:** Thermal properties of synthesized PSAN formulations.

Sample		AN	PSAN_0	PSAN_1	PSAN_3	PSAN_4	PSAN_5	PSAN_6
**T_10%_^1^**	**[%]**	218.27	213.75	181.21	213.77	208.88	206.86	207.47
**T_max1_^2^**		260.77	250.63	195.23	247.08	253.33	238.13	240.84
**T_max2_^2^**		-	624.88	474.92	594.48	626.72	590.93	562.39.
**T_max3_^2^**		-	-	-	-	-	-	801.78

**^1^** Decomposition onset temperature (measured at 10% weight loss); **^2^** the maximum decomposition temperatures, corresponding to the maximum of DTG peaks.

**Table 5:** Loading density values for the developed formulations.

**Sample**	**GP0**	**GP1**	**GP2**	**GP3**	**GP4**	**GP5**
**ρ (g/cm^3^)**	1.29	1.39	1.37	1.31	1.37	1.35
**Sample**	**GP015**	**GP115**	**GP215**	**GP315**	**GP415**	**GP515**
**ρ (g/cm^3^)**	1.35	1.47	1.42	1.35	1.43	1.41
**Sample**	**GP030**	**GP130**	**GP230**	**GP330**	**GP430**	**GP530**
**ρ (g/cm^3^)**	1.43	1.53	1.49	1.45	1.47	1.45

**Table 6:** Burning rates for each composition recorded at ambient pressure.

**Sample**	(mm/s)	**GP0**	**GP1**	**GP2**	**GP3**	**GP4**	**GP5**
v_catm_		0.70	0.72	0.69	0.71	0.73	0.72
**Sample**		**GP015**	**GP115**	**GP215**	**GP315**	**GP415**	**GP515**
v_catm_		1.01	1.03	1.04	1.07	1.03	1.06
**Sample**		**GP030**	**GP130**	**GP230**	**GP330**	**GP430**	**GP530**
v_catm_		1.15	1.19	1.16	1.14	1.10	1.17

**Table 7:** Thermal characteristics of “green” considered fuel formulations.

	Peak	Evaluation Results (°C)											
		P1 -Endothermic-			P2 -Endothermic-			P3 -Endothermic-			P4 -Exothermic-		
Sample		Start	Onset	Top	Start	Onset	Top	Start	Onset	Top	Start	Onset	Top
**GP0**		110.78	111.77	115.12	122.13	122.53	125.98	140.08	141.75	144.25	227.35	243.17	243.51
**GP1**		112.78	113.53	117.32	122.43	125.58	126.78	141.28	143.55	147.65	208.23	216.62	216.92
**GP2**		105.88	108.77	110.32	121.72	122.59	127.08	130.71	131.59	134.65	196.83	207.97	208.01
**GP3**		106.68	109.17	112.11	119.34	124.83	124.91	-	-	-	228.97	251.48	251.83
**GP4**		110.65	112.94	117.91	153.98	154.49	162.63	-	-	-	240.76	241.39	245.63
**GP5**		99.88	100.71	104.01	120.49	121.45	125.01	141.78	142.09	142.99	232.11	252.70	252.91
**GP015**		110.08	111.17	115.22	121.13	122.13	126.98	140.12	141.85	144.35	219.58	235.18	235.28
**GP115**		112.18	112.87	116.92	120.73	122.23	125.18	140.08	143.55	143.85	205.98	211.83	211.91
**GP215**		117.78	118.71	119.92	122.09	123.79	126.28	131.11	131.79	133.05	194.40	207.46	207.86
**GP315**		107.25	109.24	112.12	119.93	120.78	124.73	-	-	-	228.30	241.81	241.89
**GP415**		109.61	113.33	118.50	154.01	154.84	162.26	-	-	-	209.61	221.15	221.26
**GP515**		112.22	114.22	117.71	121.74	122.32	125.42	139.54	140.68	144.86	212.58	224.43	224.71
**GP030**		109.88	110.97	116.02	121.13	122.13	126.98	141.05	144.35	147.25	209.98	216.55	216.76
**GP130**		110.58	111.89	114.72	118.73	120.83	122.78	141.08	143.85	142.65	200.88	208.51	208.60
**GP230**		117.08	118.21	119.34	121.89	122.19	124.28	131.19	131.45	133.78	190.76	206.58	206.73
**GP330**		107.71	109.64	112.53	120.98	122.78	125.43	-	-	-	227.38	232.97	233.46
**GP430**		110.11	113.05	118.14	153.86	154.55	168.86	-	-	-	208.41	216.45	216.58
**GP530**		99.88	100.71	103.11	120.38	121.07	125.06	138.15	142.11	143.52	207.03	214.97	215.01

**Table 8:** Caloric and volume results of the “green” rocket propellant formulations.

**Sample**	**GP0**	**GP1**	**GP2**	**GP3**	**GP4**	**GP5**
Q_v_ (KJ/Kg)	4531.10	4815.99	4730.14	4775.99	4313.87	4735.61
V_sp_ (l/Kg)	702.23	632.09	637.19	629.82	690.25	648.27
**Sample**	**GP015**	**GP115**	**GP215**	**GP315**	**GP415**	**GP515**
Q_v_ (KJ/Kg)	4969.29	5085.90	5028.08	4985.11	4092.18	5110.63
V_sp_ (l/Kg)	632.51	586.41	591.14	584.14	640.34	591.32
**Sample**	**GP030**	**GP130**	**GP230**	**GP330**	**GP430**	**GP530**
Q_v_ (KJ/Kg)	6270.81	6514.99	6264.32	6381.72	6024.70	6359.59
V_sp_ (l/Kg)	574.15	554.32	567.23	571.41	590.25	560.51
**Sample**	**AP/HTPB** **propellant**	Q_v_ (KJ/Kg)	V_sp_ (l/Kg)	**DB** **propellant**	Q_v_ (KJ/Kg)	V_sp_ (l/Kg)
		7821	557		4520	820

**Table 9:** Mechanical sensitivity to friction stimuli of “green” formulations.

**Sample**	**GP0**	**GP1**	**GP2**	**GP3**	**GP4**	**GP5**
**Load Force [N]**	360	360	360	360	360	360
**Reaction Developed**	Large black smear left on the friction surfaces	Large black smear left on the friction surfaces	Large black smear left on the friction surfaces	Large black smear left on the friction surfaces	Large black smear left on the friction surfaces	Large black smear left on the friction surfaces
**Sample**	**GP015**	**GP115**	**GP215**	**GP315**	**GP415**	**GP515**
**Load Force [N]**	360	360	360	360	360	360
**Reaction Developed**	Large black smear left on the friction surfaces	Large black smear left on the friction surfaces	Large black smear left on the friction surfaces	Large black smear left on the friction surfaces	Large black smear left on the friction surfaces	Large black smear left on the friction surfaces
**Sample**	**GP030**	**GP130**	**GP230**	**GP330**	**GP430**	**GP530**
**Load Force [N]**	360	360	360	360	360	360
**Reaction Developed**	Large black smear left on the friction surfaces	Large black smear left on the friction surfaces	Large black smear left on the friction surfaces	Large black smear left on the friction surfaces	Large black smear left on the friction surfaces	Large black smear left on the friction surfaces

**Table 10:** Mechanical sensitivity to impact stimuli of “green” formulations.

**Sample**	**GP0**	**GP1**	**GP2**	**GP3**	**GP4**	**GP5**
**Impact Sensitivity [J]**	3	3	3	4	4	4
**Sample**	**GP015**	**GP115**	**GP215**	**GP315**	**GP415**	**GP515**
**Impact Sensitivity [J]**	4	4	4	4	4	4
**Sample**	**GP030**	**GP130**	**GP230**	**GP330**	**GP430**	**GP530**
**Impact Sensitivity [J]**	4	4	4	4	4	4

**Table 11:** Vacuum stability test results for the composite formulations.

**Sample**	**GP0**	**GP1**	**GP2**	**GP3**	**GP4**	**GP5**
V_deg_ (cm^3^/g)	0.775	0.712	0.798	0.801	0.783	0.767
**Sample**	**GP015**	**GP115**	**GP215**	**GP315**	**GP415**	**GP515**
V_deg_ (cm^3^/g)	0.902	0.915	0.895	0.967	0.932	0.953
**Sample**	**GP030**	**GP130**	**GP230**	**GP330**	**GP430**	**GP530**
V_deg_ (cm^3^/g)	1.010	0.996	0.985	1.005	0.981	0.997

## Data Availability

Not applicable.
